# DISMS2: A flexible algorithm for direct proteome- wide distance calculation of LC-MS/MS runs

**DOI:** 10.1186/s12859-017-1514-2

**Published:** 2017-03-03

**Authors:** Vera Rieder, Bernhard Blank-Landeshammer, Marleen Stuhr, Tilman Schell, Karsten Biß, Laxmikanth Kollipara, Achim Meyer, Markus Pfenninger, Hildegard Westphal, Albert Sickmann, Jörg Rahnenführer

**Affiliations:** 1 Department of Statistics, TU Dortmund University, Dortmund, Germany; 2 Leibniz-Institut für Analytische Wissenschaften - ISAS - e.V., Dortmund, Germany; 3 Leibniz Center for Tropical Marine Ecology (ZMT), Bremen, Germany; 4 Biodiversity and Climate Research Centre, Senckenberg Gesellschaft für Naturforschung, Frankfurt, Germany; 5 Faculty of Biological Science, Institute for Ecology, Evolution and Diversity, Department of Molecular Ecology, Goethe University, Max-von-Laue-Straße 9, Frankfurt am Main, 60438 Germany; 6 Department of Chemistry, College of Physical Sciences, University of Aberdeen, Aberdeen, Scotland, United Kingdom; 7 Medizinische Fakultät, Medizinisches Proteom-Center (MPC), Ruhr-Universität Bochum, Universitätsstraße 150, Bochum, 44801 Germany

**Keywords:** Proteomics, LC-MS/MS, Mass spectrometry, Comparison of MS/MS spectra, Peptide identification, Distance of LC-MS/MS runs

## Abstract

**Background:**

The classification of samples on a molecular level has manifold applications, from patient classification regarding cancer treatment to phylogenetics for identifying evolutionary relationships between species. Modern methods employ the alignment of DNA or amino acid sequences, mostly not genome-wide but only on selected parts of the genome. Recently proteomics-based approaches have become popular. An established method for the identification of peptides and proteins is liquid chromatography-tandem mass spectrometry (LC-MS/MS). First, protein sequences from MS/MS spectra are identified by means of database searches, given samples with known genome-wide sequence information, then sequence based methods are applied. Alternatively, de novo peptide sequencing algorithms annotate MS/MS spectra and deduce peptide/protein information without a database. A newer approach independent of additional information is to directly compare unidentified tandem mass spectra. The challenge then is to compute the distance between pairwise MS/MS runs consisting of thousands of spectra.

**Methods:**

We present DISMS2, a new algorithm to calculate proteome-wide distances directly from MS/MS data, extending the algorithm compareMS2, an approach that also uses a spectral comparison pipeline.

**Results:**

Our new more flexible algorithm, DISMS2, allows for the choice of the spectrum distance measure and includes different spectra preprocessing and filtering steps that can be tailored to specific situations by parameter optimization.

**Conclusions:**

DISMS2 performs well for samples from species with and without database annotation and thus has clear advantages over methods that are purely based on database search.

**Electronic supplementary material:**

The online version of this article (doi:10.1186/s12859-017-1514-2) contains supplementary material, which is available to authorized users.

## Background

In recent years proteomics has become of great interest in biochemistry. New methods and technologies are constantly being developed [1, 2]. Qualitative and quantitative proteomics strategies are particularly useful to analyze samples measured under different conditions or samples from different phenotypes. Another application was presented by Palmblad and Deelder [3] who reconstructed the unique correct phylogenetic tree for the great apes and other primates based solely on proteome-wide measurements. Furthermore, Yilmaz et al. [4] have recently proposed a pipeline for differential proteomics in unsequenced species.

Most of the workflows in proteomics nowadays are based on mass spectrometry [5–7], replacing 2D gel electrophoresis. A great variety of instruments is being used and refined. Dealing with the high complexity of protein or peptide samples, liquid chromatography as separation technique is often combined with mass spectrometry. Tandem mass spectrometry, MS/MS, involves at least two stages of mass analysis and an intermediate fragmentation step. MS/MS spectra comprise of detected intensities of occurring masses corresponding to peptide fragments.

The identification of peptides and proteins using MS and MS/MS data is typically performed by database-dependent search algorithms, e.g., with Mascot [8]. Peptide sequences are verified by large and continuously updated databases that are derived from genome sequencing projects. These databases are usually well curated and often manually annotated. However, novel peptides nonexistent in databases cannot be identified with this approach. De novo peptide sequencing algorithms [6, 9, 10] are independent of database searches, but robust error estimation strategies are still lacking.

By omitting the peptide identification step mass spectra from different samples can be directly compared. Palmblad and Deelder [3] introduced a basic algorithm differentiating between blood samples. Two components are required for comparing samples on a proteome-wide scale. First, appropriate distance measures for mass spectra are needed [6, 11, 12]. Second, the information of thousands of spectra has to be aggregated to compute the global distance between pairwise LC-MS/MS runs.

Our main goal is to provide a general method for the comparison of different samples using data of LC-MS/MS runs. As a proof of concept, our new algorithm DISMS2 is applied to real data of LC-MS/MS runs. This includes both known species with established protein databases as well as two organisms with no prior comprehensive genomics and proteomics studies, namely *Radix* and *Amphistegina*, for which only direct spectra comparisons are feasible.

The resulting distances of DISMS2 are compared to a standard database search with Mascot evaluating the competitiveness of our flexible approach.

## Methods

We introduce the new flexible DISMS2 algorithm that calculates explicit distances between pairs of LC-MS/MS runs. First, a collection of 27 MS/MS runs from different species is presented. Second, a conventional Mascot database search as competitive method is explained. Third, an overview of appropriate distance measures between single spectra is given. Then the algorithm DISMS2 is explained in detail, and finally an approach for parameter optimization for DISMS2 is presented.

### Samples and LC-MS/MS analysis

Proteolytic (tryptic) digests of five sequenced organisms, i.e. (i) human (*Homo sapiens*, H, HeLa cell line), (ii) mouse (*Mus musculus*, M, C2C12 cell line), (iii) yeast (*Saccharomyces cerevisiae*, Y), (iv) roundworm (*Caenorhabditis elegans*, C), and (v) fruit fly (*Drosophila melanogaster*, D) and of four organisms without sequenced genome, i.e. (vi) fresh water snail *Radix* species: molecular operational taxonomic unit (MOTU) 2 (R2), 4 (R4) and (vii) foraminifera species *Amphistegina lessonii* (Al), *Amphistegina gibbosa* (Ag) were analyzed using an Ultimate 3000 nano RSLC system coupled to a Q Exactive HF mass spectrometer (both Thermo Scientific). Each sample was measured in triplicate (1 μg each) resulting in a dataset of 27 MS/MS runs. The samples were analyzed in randomized order to minimize systematic errors. Detailed information about sample preparation and LC-MS/MS analyses is provided in a document about Material and Methods [see Additional file 1].

### Database search

MS data interpretation was conducted using Proteome Discoverer 1.4 (Thermo Scientific) and Mascot 2.4 (Matrix Science). Database searches of the five model organisms (i.e. human, mouse, yeast, roundworm and fruit fly) were performed in a target/decoy mode against their respective protein sequence (FASTA) databases [see Additional file 1]. Trypsin was selected as enzyme, and two missed cleavage sites were allowed. Carbamidomethylation of cysteine was set as fixed and oxidation of methionine was set as dynamic modifications. MS and MS/MS tolerances were set to 10 ppm and 0.02 Da respectively, and only peptide-to-spectrum matches (PSMs) with search engine rank 1 and a false discovery rate (FDR) <1*%* (Percolator setting) were considered.

### Distance measures

For any MS/MS run *i* containing *n*_*i*_ MS2 spectra define spectrum $S_{k_{i}}$ with rank *k* in run *i* as a set of two vectors $\boldsymbol {x}_{k_{i}}$ and $\boldsymbol {I}_{k_{i}}$ with length $p_{k_{i}}$:

$${} S_{k_{i}} = \left\{\boldsymbol{x}_{k_{i}}, \boldsymbol{I}_{k_{i}}\right\} = \left\{\left(x_{k_{i},1},\ldots,x_{k_{i},p_{k_{i}}}\right)', \left(I_{k_{i},1},\ldots,I_{k_{i},p_{k_{i}}}\right)' \right\} $$

The m/z (mass-to-charge) ratios $\boldsymbol {x}_{k_{i}}$ are sorted in ascending order, and corresponding peak intensities are labeled with $\boldsymbol {I}_{k_{i}}$.

According to the resolution of the experiment the range of m/z values can be subdivided into small intervals so that every peak can be assigned to exactly one interval. Then an alternative definition of spectrum $S_{k_{i}}$ is a vector $\tilde {\boldsymbol {I}}_{k_{i}} = (\tilde {I}_{k_{i},1},\ldots,\tilde {I}_{k_{i},\tilde {p}})'$ with $\tilde {p}$ entries, where the entry at a specific position is the peak intensity, if one peak was assigned, and otherwise 0.

The most commonly used distance measure for the pairwise comparison of mass spectra is the cosine distance *d*_cos_ [11]. For a pair of vectors of intensities $\tilde {\boldsymbol {I}}_{k_{i}}$ and $\tilde {\boldsymbol {I}}_{l_{j}}$, the cosine similarity of the spectra *k*_*i*_ and *l*_*j*_ is the ratio of the dot product and the product of the Euclidean norms of the intensity vectors, according to the alternative definition. The cosine distance *d*_cos_ is then calculated by subtracting the cosine similarity from 1:

$$\begin{array}{*{20}l} d_{\text{cos}}(S_{k_{i}}, S_{l_{j}}) &= 1 - \frac{\left\langle \tilde{\boldsymbol{I}}_{k_{i}}, \tilde{\boldsymbol{I}}_{l_{j}} \right\rangle}{\left| \tilde{\boldsymbol{I}}_{k_{i}} \right| \left| \tilde{\boldsymbol{I}}_{l_{j}} \right|}\\ &= 1 - \frac{\sum_{q=1}^{\tilde{p}}{\tilde{I}_{k_{i},q} \cdot \tilde{I}_{l_{j},q}}}{\sqrt{\sum_{q=1}^{\tilde{p}}{\tilde{I}_{k_{i},q}^{2}}} \cdot \sqrt{\sum_{q=1}^{\tilde{p}}{\tilde{I}_{l_{j},q}^{2}}}} \end{array} $$

Depending on preprocessing of the spectra, e.g. only considering the top topn (topn ∈N) peaks of each spectrum, a cosine distance that neglects intensities, is appropriate. Novak and Hoksza [12] have introduced the angle distance, a distance corresponding to cosine distance, with the original spectrum definition given by:

$$\begin{array}{*{20}l}{} d_{\text{angle}}&(S_{k_{i}}, S_{l_{j}}, \epsilon)\\ &= \arccos\left(\frac{\sum_{q=1}^{p_{k_{i}}}{\max_{q^{*}=1,\ldots,p_{l_{j}}}{ 1_{\left\{\left|x_{k_{i},q} - x_{l_{j},q^{*}}\right| \leq \epsilon \right\}} }}}{\sqrt{p_{k_{i}} \cdot p_{l_{j}}}}\right) \end{array} $$

Several other distance measures have been discussed, such as Pearson’s or Spearman’s correlation [11]. Novak and Hoksza [12] have introduced the Parametrized Hausdorff distance $d_{\text {PH}}(S_{k_{i}}, S_{l_{j}}, \delta, k) = \max (h(S_{k_{i}}, S_{l_{j}}, \delta, k), h(S_{l_{j}}, S_{k_{i}}, \delta, k))$ with

$$\begin{array}{*{20}l}{} h&(S_{k_{i}}, S_{l_{j}}, \delta, k)\\ {}&= \frac{1}{p_{k_{i}}} \sum_{q=1}^{p_{k_{i}}}{\left(\min_{q^{*}=1,\ldots,p_{l_{j}}}{ \left|x_{k_{i},q} - x_{l_{j},q^{*}}\right|} 1_{\left\{\left|x_{k_{i},q} - x_{l_{j},q^{*}}\right| > \delta \right\}} \right)^{1/k}. } \end{array} $$

Given an error tolerance *δ*, *h* averages the k-th root of the minimal absolute distance greater than *δ* of the position of all peaks of spectrum $S_{k_{i}}$ compared to all peaks of spectrum $S_{l_{j}}$.

### DISMS2

The pseudo code of our new algorithm DISMS2 is shown in Algorithm 1. Calculating the pairwise **DIS**tances of *N* MS/MS (**MS2**) runs is a four-step procedure, consisting of spectra filtering, checking constraints for matching, matching of MS2 spectra, and calculation of the distance matrix with pairwise distances of MS/MS runs. The algorithm has been implemented in the statistical programming language R [13].



The first step is preprocessing and filtering of MS/MS spectra. Additionally it can be specified if only peaks of MS2 spectra with top topn highest intensities are included in the analysis. Then all spectra are binned with a flexible binsize bin. Binning with a fixed binsize bin=0.2 has been applied in compareMS^2^ [3]. All intensities with m/z ratio *x* in a small interval [*n* ^∗^·bin,(*n* ^∗^+1)·bin) (*n* ^∗^∈N_0_) are replaced by one representative, the maximum intensity at the central m/z ratio value (*n* ^∗^+0.5)·bin.

The concept of the procedure is to match all MS/MS spectra in run *i* with the most similar spectra in run *j* and vice versa. Due to a long computing time and for reasons of content the number of possible matching candidates is reduced by checking three constraints in step 2.

Constraints (a)–(c) are checked in the following order. First, only spectra with a similar retention time are considered. The usage of a HPLC before MS analysis justifies the constraint of a similar retention time since peptides with the same properties elute from the column at the same time. While retention time alignment is necessary for an optimal analysis, the following method is fast and adequate for filtering. To save computing time, instead of determining a time window the tolerance ret limits the number of MS/MS spectra in run *j* before and after any MS/MS spectrum in run *i* by the rank of the scan number. By filtering all candidate spectra with rank *l* in an interval [*k*−ret,*k*+ret] for matching with spectrum *k* in run *i*, at most 2·ret+1 MS2 spectra remain. The tolerance ret can be increased to ensure that no best matching spectra are missed.

Although mass to charge ratios are displayed to allow for comparisons of spectra with different charge states, spectra might look different due to different properties. For this reason constraint (b) guarantees that only matches of spectra with the same precursor charge state are considered. Again the number of possible candidates is reduced.

Apparently peptides with the same amino acid sequences and the same post-translational modifications have same masses. This leads to constraint (c) allowing only the comparison of spectra with similar precursor masses. For any spectrum *k* in run *i* with precursor mass $m_{k_{i}}$ only spectra *l* of run *j* inside a small interval $\left [m_{k_{i}} \cdot (1-10^{-6}\text {\texttt {prec}}), m_{k_{i}} \cdot (1+10^{-6}\text {\texttt {prec}})\right ]$ are considered. This ensures a maximum precursor mass accuracy of prec ppm (parts per million): $|(m_{l_{j}} - m_{k_{i}})/m_{k_{i}}|\cdot 10^{6} \leq \text {\texttt {prec}}$. If no spectrum of run *j* fulfills all constraints for spectrum *k* in run *i*, no match is available. Then spectrum *k* is assigned a distance greater than a threshold cdis.

Finally in step 4 sequentially for each spectrum *k* in run *i* distances dist to all remaining candidate spectra in run *j* are calculated. As mentioned above several appropriate distance measures can be chosen, for example the most commonly used cosine distance *d*_cos_, the angle distance *d*_angle_, or the parametrized Hausdorff distance *d*_PH_. Depending on the chosen distance measure dist a binary cut-off threshold cdis for a hit, i.e. same or different spectrum, has to be fixed. For the cosine distance *d*_cos_ Palmblad and Deelder [3] have shown that cdis=0.2 is a reasonable choice. A distance *d* ^∗^(*i*,*j*) between runs *i* and *j* is the frequency of spectra in run *i* with no match (all distances greater than cdis) in run *j*. Match means that the spectrum in run *j* with smallest distance dist is considered and this minimal distance is smaller than cdis. The distance *d* ^∗^ is not symmetric because the process is directed. Thus the procedure is repeated with exchanged runs *i* and *j* and finally the mean *d*(*i*,*j*)=(*d* ^∗^(*i*,*j*)+*d* ^∗^(*j*,*i*))/2 of the directed distances is calculated. *N*·(*N*−1) directed distances *d* ^∗^ have to be calculated to fill the distance matrix *d*.

### Parameter optimization for DISMS2

In our new algorithm DISMS2 several parameters have to be set and therefore an appropriate parameter optimization is needed. Given the data as mentioned above, a distance matrix between runs with size 27×27 is computed using DISMS2. Distances within groups of technical replicates of organisms should be smaller than distances between different organisms. However, due to the random selection of precursor ions for MS/MS analysis, data dependent acquisition (DDA) is biased to the most abundant peptides present in a complex sample. Further, in DDA mode the intra-sample variation of peptide identifications between technical replicates is high (≥50*%*) [14]. For this reason, an ANOVA like approach for distance matrices, adonis (R package vegan [15]), is used for evaluation. Anderson [16] has introduced this non-parametric multivariate analysis of variance which is applicable for a distance matrix explaining different sources of variation. Variations of distances are divided into two parts, one representing variation of technical replicates within species and the other variation between species. A permutation test (by default with 10 000 permutations) with pseudo-F ratio between the mean sum of squares of distances between and within species is used. As goodness-of-fit measure the partial R-squared between groups of technical replicates, i.e. the ratio of sum of squares of distances between species and the sum of squares of all distances, is used. Values close to 1 are desired.

## Results and discussion

First, the application of the new algorithm DISMS2 on real data is presented in detail, including data preprocessing and choice of parameters. Second, DISMS2 is compared to a common Mascot database search (on spectra and peptide level). Finally, distances between species are visualized by dendrograms using average linkage hierarchical clustering.

### Application of DISMS2 on real data with parameter optimization

We implemented DISMS2 in R [13] and applied it on 27 MS/MS runs to compare samples of human, mouse, yeast, roundworm, fruit fly, two different *Radix* species and two different foraminifera species. The ProteoWizard tool MSConvertGUI [17] was used to convert Thermo RAW files into mzXML files. The open data format mzXML can be read with the R package readMzXmlData [18].

To find appropriate parameter settings in DISMS2 we used a full factorial design. Due to time and memory costs the number of parameter combinations was limited. The values of factor levels in design 1 were set according to preliminary investigations and expert advice (see Table 1). Especially the accepted precursor mass shift prec is set constant (10 ppm). Since the angle distance is not bounded in the interval [ 0,1], in design 2 more factors were added with higher values for the cutoff cdis. In total 81 factor combinations were compared by means of the partial R squared as goodness of fit measure, based on adonis, an ANOVA like approach for distance matrices.

**Table 1:** Design of factor levels of parameters in evaluation of DISMS2. All 81 different combinations of all parameter values, 72 combinations for design 1, and 9 combinations for design 2

Parameter	Description	Design 1	Design 2
topn	topn highest peaks, *∞* means no selection	20, 50, *∞*	20, 50, *∞*
bin	binning with binsize bin	0.01, 0.2	0.2
ret	size of retention time window	1000, 3000	3000
prec	accepted precursor mass shift (ppm)	10	10
dist	distances between spectra	*d*_angle_(*ε*=0.05),*d*_cos_,	*d*_angle_(*ε*=0.05)
		*d*_PH_(*δ*=0.05;*k*=50)	
cdis	cutoff for distance of spectra	0.1, 0.3	0.4, 0.5, 0.6

The results of the parameter optimization are summarized in Table 2. In particular distance measures are of great interest, so bold printed lines correspond to the optimized parameters for the different distance measures.

**Table 2:** Results of parameter optimization for DISMS2 with partial R-squared (adonis)

Rank	topn	bin	ret	dist	cdis	partial R-squared
1	*∞*	**0.20**	**3000**	*d*_cos_	**0.3**	**0.923**
2	50	0.20	3000	*d*_cos_	0.3	0.923
3	20	0.20	3000	*d*_cos_	0.3	0.923
4	*∞*	0.20	3000	*d*_cos_	0.1	0.892
5	50	0.20	3000	*d*_cos_	0.1	0.892
6	20	0.01	3000	*d*_cos_	0.3	0.890
7	20	0.20	3000	*d*_cos_	0.1	0.890
8	50	0.01	3000	*d*_cos_	0.3	0.890
9	*∞*	0.01	3000	*d*_cos_	0.3	0.890
**10**	**20**	**0.01**	**3000**	*d*_PH_	**0.3**	**0.879**
11	*∞*	0.20	1000	*d*_cos_	0.3	0.878
⋮	⋮	⋮	⋮	⋮	⋮	⋮
**23**	**20**	**0.20**	**3000**	*d*_angle_	**0.6**	**0.808**
⋮	⋮	⋮	⋮	⋮	⋮	⋮
81	*∞*	0.20	3000	*d*_angle_	0.3	0.308

Accepted precursor mass shift constant (prec = 10 ppm)

Bold printed lines correspond to the optimized parameters for the different distance measures

We requested 3GB RAM on one core of an eight-core Intel Xeon E5-2630 (2.4 GHz, 128 GB RAM, Debian Linux 8.3.0 operating system). The median runtime of DISMS2 for 27 MS/MS runs for one of in total 81 factor combinations (settings) is 13.41 hrs (range 4.49 - 20.01 hrs). For the best ranked setting (see Table 2) 15.73 hrs are needed. The use of constraints (a) – (c) in step 2 of Algorithm 1, especially an appropriate choice of retention time tolerance ret, drastically reduces the runtime of DISMS2. Increasing ret for the best ranked setting from 3000 to 40000 leads to a runtime of more than 26 hrs, an increase by a factor of 1.65. Removing all constraints would result in a dramatic runtime increase in the range of 2 years. The use of constraints leads to a small number of candidates for matches. In case of H1 and H2, for example, on average only 2.5 out of more than 35000 candidates remain.

**Fig. 1:**
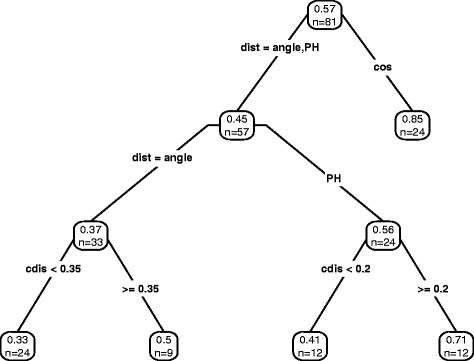
Regression tree fitted to explain the partial R-squared (adonis) based on combinations of parameter settings. Different Parameter settings of topn, bin, ret, dist, and cdis were used. Each node displays the average partial R-squared in the node (*top*) and the number of observations that fall in the node (*down*). Classification was performed using the statistical programming language R, R package rpart [19]

To quantify the influence of the parameters a regression tree was constructed (Fig. 1, [19]). The choice of the distance measure has the highest influence. The cosine distance outperforms *d*_PH_ and *d*_angle_. Still, the parametrized Hausdorff distance is competitive when a higher cutoff is chosen.

**Fig. 2:**
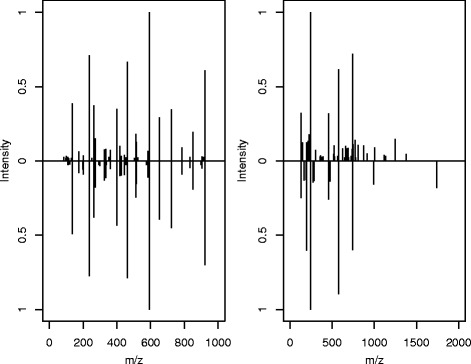
Comparison of two similar (*left*) and two diverse (*right*) spectra. Preprocessing (topn=20, bin= 0.01) was used and parameters for distance measures are as in Table 1. Distances for left example: *d*_cos_=0.010, *d*_angle_=0.451, *d*_PH_=0.105, distances for right example: *d*_cos_=0.396, *d*_angle_=1.204, *d*_PH_=0.746

The impact of the choice of a distance measure for spectra is shown in Fig. 2 illustrating two similar and two diverse spectra, respectively. The value of the angle distance in case of two similar spectra is almost as large as the value of the cosine distance in case of two distinct spectra.

### Comparison of DISMS2 with state of the art database search

To evaluate the quality of DISMS2 we compared it to database search algorithms. DISMS2 requires a filter check, whereas database search requires an annotation check. We analyzed several methods combining algorithmic steps in different ways, see Table 3 for a list of all compared algorithms.

**Table 3:** Overview over all algorithms compared

Name	Search	Spectrum	Annotation	Filter	Duplicates
	method	universe	check	check	
DB.ra	Database	Reduced	Yes	No	Kept
DB.ra.nodup	Database	Reduced	Yes	No	Removed
DISMS2.f	Distance	All	No	Yes	Kept
DB.a	Database	All	Yes	No	Kept
DISMS2.af	Distance	All	Yes	Yes	Kept
DB.af	Database	All	Yes	Yes	Kept

For all algorithms we calculated the average relative number of no hits of a directed search. The general principle is that a list of spectra associated with an MS/MS run is compared one by one with a second list of candidate spectra associated with a second MS/MS run. The algorithms differ in the definition of a hit, the spectrum universe, possible annotation and filter checks, and potential removal of duplicates.

Search method *database* means that a hit is counted if in the list of candidate spectra the same peptide is annotated. For the search method *distance* a hit means that the distance between the spectrum and a candidate spectrum is smaller than cdis = 0.3.

The spectrum universe is the union of all MS/MS spectra in respective runs. If *all* spectra are included in the analysis complete runs are used. A *reduced* spectrum universe stands for a selection of MS/MS spectra with peptide annotations.

Annotation check means that for matching candidates both spectra have to be annotated by a Mascot peptide-to-spectrum-match. Possibly matching candidates are downgraded as no hit. For about 30 to 60% of all spectra (on average for roundworm 29%, human 34%, mouse 40%, fruit fly 45% and yeast 57%) peptide hits are missing, i.e. the database search with Mascot resulted in no hit, meeting a 1*%* FDR criterion.

Filtering means that for matching candidates the constraints for retention time, precursor mass and charge state have to be fulfilled (see step 3 in DISMS2).

Usually duplicated peptide annotations are *kept* meaning that all spectra are considered in the spectrum universe. *Removing* duplicates (nodup) of peptide annotations means that a hitlist of all peptides annotated at least once is used.

We now describe all compared algorithms listed in Table 3. The methods DISMS2.f and DB.ra are the default versions. DISMS2.f uses filtering and considers *all* spectra, whereas DB.ra uses no filtering and considers only spectra that are annotated with a peptide sequence. In DB.ra.nodup, additionally multiple spectra annotated with the same peptide are replaced by one representative (*removal* of duplicates).

Search method *distance* is associated with a filter check whereas search method *database* includes an annotation check. The differences between DISMS2.f and DB.a are only the annotation check and the filter check. All spectra are included in the spectrum universe of DB.a, in contrast to DB.ra with a reduced universe.

For a meaningful comparison we also considered two algorithms with both filtering and annotation checks. Including all MS/MS spectra in the spectrum universe, DISMS2.af and DB.af only differ by the search method.

**Fig. 3:**
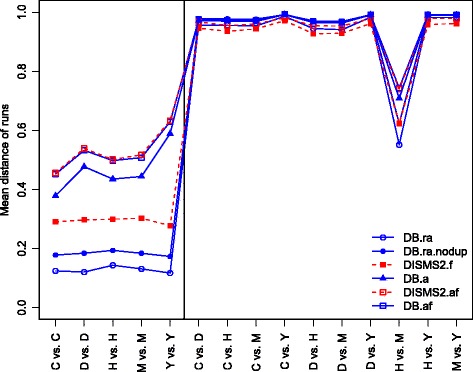
Mean relative number of partners for different ways of proteome comparisons methods. Steps of search method, spectrum universe, annotation check, filter check and potential removal of duplicates in different ways between (*right*) and within (*left*) species of roundworm (C), fruit fly (D), human (H), mouse (M) and yeast (Y) are compared

The mean distances of runs are computed for all methods between and within species, as shown in Table 4 and in Fig. 3. In Table S1 [see Additional file 2] the total number of MS/MS spectra for different species comparisons is shown in detail. Corresponding standard errors indicating variations of technical replicates are negligibly small, see Table S2 [see Additional file 3]. In comparisons within species standard errors are smaller than 0.008, and between species at most 0.002.

**Table 4:** Mean distances of runs for different ways of proteome comparisons methods between and within species

Method	DB.ra	DB.ra.nodup	DISMS2.f	DB.a DISMS2.af	DB.af	
C vs. C	0.125	0.178	0.290	0.379	0.456	0.452
D vs. D	0.121	0.184	0.297	0.477	0.539	0.533
H vs. H	0.143	0.194	0.299	0.435	0.501	0.498
M vs. M	0.131	0.184	0.303	0.444	0.518	0.508
Y vs. Y	0.117	0.173	0.278	0.589	0.633	0.629
C vs. D	0.957	0.978	0.946	0.972	0.967	0.976
C vs. H	0.956	0.978	0.935	0.970	0.955	0.973
C vs. M	0.954	0.977	0.944	0.969	0.961	0.973
C vs. Y	0.986	0.992	0.972	0.992	0.981	0.993
D vs. H	0.945	0.971	0.927	0.966	0.954	0.969
D vs. M	0.941	0.969	0.929	0.963	0.953	0.967
D vs. Y	0.983	0.992	0.962	0.991	0.979	0.992
H vs. M	0.551	0.623	0.624	0.709	0.740	0.744
H vs. Y	0.982	0.991	0.958	0.990	0.979	0.991
M vs. Y	0.982	0.991	0.962	0.990	0.979	0.991

For different ways of proteome comparisons methods steps of search method, spectrum universe, annotation check, filter check and potential removal of duplicates between are combined. Mean distances of runs between (down) and within (top) species of roundworm (C), fruit fly (D), human (H), mouse (M) and yeast (Y) are shown

The mean distances are small within species and large between species. DB.ra and DB.ra.nodup generate smaller values, followed by DISMS2.f. DB.a is not able to keep up. Largest distances are generated for DB.af and DISMS2.af. It should be noted that the exact composition of peptides of analyzed samples is unknown as database annotation might be incorrect or incomplete. Thus the interpretation of larger or smaller values might be imprecise.

For the algorithms DB.ra and DB.ra.nodup the distances are calculated based on a reduced spectrum universe so that many spectra with low quality have been removed. Thus it is expected that many spectra without annotation implying large distances are removed, which leads to smallest values within species smaller than 20% in Table 4.

Since DB.af uses the same filtering as DISMS2.af, results are directly comparable. Annotation and filtering checks reduce the number of possible matches. As aforementioned missing annotations in list 1 have a share of 30 to 60%. Furthermore for 8 to 13% (within species) and 30 to 50% (between species) of all spectra no candidate spectra are remaining that fulfill all filtering requirements and are annotated (for further details see Table S3 [see Additional file 4]).

**Fig. 4:**
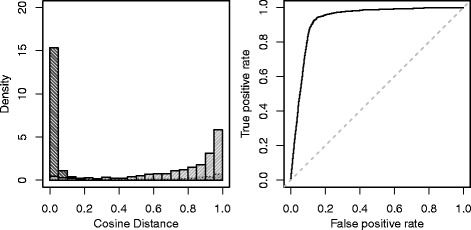
Histogram of MS/MS spectra distances and corresponding ROC curve. Histogram (*left*) showing MS/MS spectra distances of spectra pairs with the same peptide annotation in DB.af (*dark gray*) and of the remaining spectra pairs with different peptide annotation in DB.af (*light gray*), for H1 vs. H2. ROC curve (*right*) showing performance of cosine distance as a binary classifier of same or different peptides for different thresholds cdis

In case of same annotated peptides the cosine distance is typically small, see Fig. 4 (left, dark gray) with the distribution of the cosine distance of MS/MS spectra for two human samples (H1 vs. H2), with mode near 0. However, if the peptide annotation of the matching spectrum is different, the situation differs (Fig. 4, left, light gray). Most of the distances are high as one might expect. Only a few values are smaller than 0.3. In these cases very similar spectra are marked as different by the annotation approach, possibly due to missing or wrong hits. Cosine distance as a binary classifier for same or different peptides is a good choice. The ROC curve (Fig. 4, right) displays the performance, plotting the true positive rate (TPR) against the false positive rate (FPR) for different thresholds cdis. The area under the curve is 0.93 indicating a good performance. For the chosen threshold cdis=0.3 we obtain TPR=92.3*%* and FPR=13.3*%*.

The commonly used method DB.ra.nodup differs from the other methods because duplicated measured peptides are weighted different. The mean distance within species of the usual peptide list comparison DB.ra.nodup is about 18 percent, DISMS2.f based on all spectra leads to little higher values of about 30 percent.

Most of the comparisons between different species yield values above 90%, except the comparison of human and mouse samples. Again DB.ra leads to smallest values (55.1%), followed by DB.ra.nodup (62.3%) and DISMS2.f (62.4%).

For the comparison of two methods we first calculated the absolute differences of proteome distances between the two methods and then the coefficient of variation (CV) of these values, i.e. the ratio of standard deviation and mean (see Table S4 and S5 [see Additional files 5 and 6]). 5 A value smaller than 0.5 indicates a relevant difference between the corresponding two methods. In most cases there are relevant differences. Only DISMS2.af and DB.af perform very similar, since CVs are relatively large, some of them considerably larger than 0.5.

The filtering check is needed for the search method *distance* to speed up the computing time. Exemplary for the two human samples H1 and H2 DISMS2 is computed without filtering check. The results for all methods applied to these two samples in Table 5 show that DISMS2 without filtering is competitive even to the database methods with reduced spectrum universe.

**Table 5:** Example of distance for two human MS/MS runs

Name	*d* ^∗^(H1, H2)	*d* ^∗^(H2, H1)	d(H1, H2)
DB.ra	0.1467	0.1487	0.1477
DB.ra.nodup	0.1971	0.2010	0.1990
DISMS2.f	0.2969	0.3050	0.3009
DB.a	0.4321	0.4422	0.4371
DISMS2.af	0.4968	0.5087	0.5027
DB.af	0.4935	0.5068	0.5002
DISMS2	0.1616	0.1645	0.1631

Directed cosine distances *d* ^∗^ in both directions and mean of directed distances *d* of two human MS/MS runs H1 and H2 are compared. DISMS2 stands for DISMS2.f without filtering check

### Visualization of distances between species

Dendrograms using average linkage hierarchical clustering were used as trees to illustrate distances between MS/MS runs of different samples and its technical replicates. In average linkage clustering the mean distance between all pairs of elements is used for fusion of clusters.

**Fig. 5:**
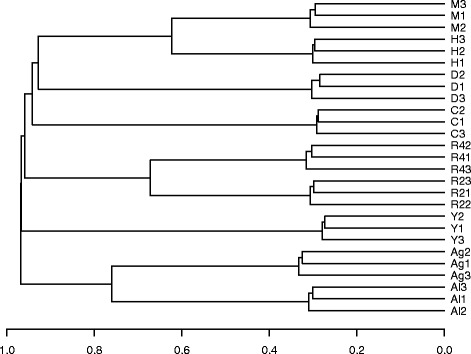
Dendrogram computed via DISMS2.f. Dendrogram for three technical replicates each of roundworm (C), fruit fly (D), human (H), mouse (M), yeast (Y), *Radix* MOTU 2 (R2), *Radix* MOTU 4 (R4), *A. gibbosa* (Ag) and *A. lessonii* (Al) using average linkage hierarchical clustering based on all pairwise distances of 27 MS/MS runs. Computed via DISMS2.f with optimized parameters: topn = *∞*, bin = 0.2, ret = 3000, prec = 10, dist = *d*_cos_ and cdis = 0.3

The first dendrogram visualizes the distance matrix constructed with DISMS2.f for all 27 MS/MS runs (Fig. 5). The average distance between technical replicates is about 30%. Nodes connecting mouse and human samples (62.4%) as well as two *Radix* species (67.2%) and foraminifera species (76.1%) indicate high similarity.

**Fig. 6:**
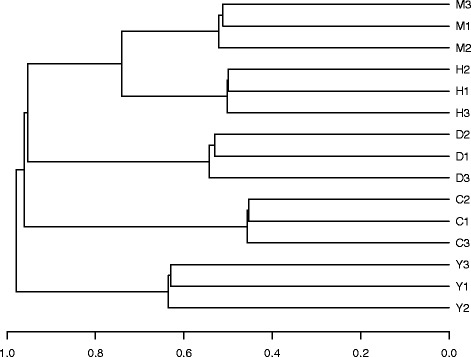
Dendrogram computed via DISMS2.af. Dendrogram for three technical replicates each of roundworm (C), fruit fly (D), human (H), mouse (M) and yeast (Y) using average linkage hierarchical clustering based on all pairwise distances of 15 MS/MS runs. Computed via DISMS2.af with optimized parameters: topn = *∞*, bin = 0.2, ret = 3000, prec = 10, dist = *d*_cos_ and cdis = 0.3

Further dendrograms (Fig. 6 and Figure S1–S4 [see Additional files 7, 8, 9 and 10]) are generated for the other methods for all 15 samples with available database annotations. The distance matrix for DB.af is almost identical to the one to DISMS2.af (Fig. 6), with notably less separation between species.

## Conclusions

DISMS2 is a new user-friendly algorithm implemented in R for the proteome-wide distance calculation of different MS/MS runs. It performs well with data from different organisms, if parameter optimization is performed. Measuring technical replicates is the basis for selecting suitable parameters, based on an ANOVA like approach. Alternatively, prior knowledge can be used to choose adequate parameters. We carefully compared DISMS2.f (with filtering) with a state of the art method, the database search Mascot DB.a. Whereas DISMS2.f uses a filtering step that requires to set a critical distance *c* *d* *i* *s*, DB.a suffers from the large number of missing database hits. For a fair comparison, DISMS2.af and DB.af use the same spectrum universe of all spectra and perform annotation and filtering checks in the same way. A crucial difference is the different handling of duplicated spectra that are often neglected in database search methods. Thus a future goal is to perform clustering of spectra [20–23] before matching of partners, in order to further improve DISMS2 and make it directly comparable to the standard DB.ra.nodup. A huge benefit of DISMS2 is its applicability to samples from species without database annotation, as demonstrated on the fresh water snail *Radix* species (molecular operational taxonomic unit (MOTU) 2 and 4) and on the foraminifera *Amphistegina* *lessonii* and *gibbosa*. Further, when performing database searches in closely related species or applying de novo approaches DISMS2 can help to validate the results.

## Additional files

Additional file 1 Materials and Methods. This file provides detailed information about the sample preparation and LC-MS/MS analyses. (PDF 145 kb)

Additional file 2
**Table S1**. Total number of MS/MS spectra in Table 4. (PDF 8 kb)

Additional file 3
**Table S2**. Standard Errors belonging to mean distances in Table 4. (PDF 9 kb)

Additional file 4
**Table S3**. Additional information to Algorithm DB.af. Mean relative number of partners (same peptide), different peptides, missing annotation in list 1 and no remaining candidates after *filtering* in list 2 in Algorithm DB.af. (PDF 9 kb)

Additional file 5
**Table S4**. Coefficients of variation (CVs) of absolute differences of distances between two proteome comparisons (database) methods. CVs of absolute differences of distances between two methods, within species (top) and between species (bottom), for the species roundworm (C), fruit fly (D), human (H), mouse (M), and yeast (Y). Values smaller than 0.5 indicate relevant differences between the corresponding method pairs. Values greater than 0.5 are marked (*). (PDF 8 kb)

Additional file 6
**Table S5**. Coefficients of variation (CVs) of absolute differences of distances between two proteome comparisons methods. CVs of absolute differences of distances between two methods, within species (top) and between species (bottom), for the species roundworm (C), fruit fly (D), human (H), mouse (M), and yeast (Y). Values smaller than 0.5 indicate relevant differences between the corresponding method pairs. Values greater than 0.5 are marked (*). (PDF 9 kb)

Additional file 7
**Figure S1**. Dendrogram for three technical replicates each of roundworm (C), fruit fly (D), human (H), mouse (M) and yeast (Y) using average linkage hierarchical clustering based on all pairwise distances of 15 MS/MS runs. Computed via method DB.ra. (PDF 4 kb)

Additional file 8
**Figure S2**. Dendrogram for three technical replicates each of roundworm (C), fruit fly (D), human (H), mouse (M) and yeast (Y) using average linkage hierarchical clustering based on all pairwise distances of 15 MS/MS runs. Computed via method DB.ra.nodup. (PDF 4 kb)

Additional file 9
**Figure S3**. Dendrogram for three technical replicates each of roundworm (C), fruit fly (D), human (H), mouse (M) and yeast (Y) using average linkage hierarchical clustering based on all pairwise distances of 15 MS/MS runs. Computed via method DB.a. (PDF 4 kb)

Additional file 10
**Figure S4**. Dendrogram for three technical replicates each of roundworm (C), fruit fly (D), human (H), mouse (M) and yeast (Y) using average linkage hierarchical clustering based on all pairwise distances of 15 MS/MS runs. Computed via method DB.af. (PDF 4 kb)

## Abbreviations

Ag: Amphistegina gibbosa
Al: Amphistegina lessonii
ANOVA: Analysis of variance
C: roundworm Caenorhabditis elegans
CV: Coefficient of variation
D: fruit fly Drosophila melanogaster
DISMS2: Algorithm for calculating pairwise distances of MS/MS runs
DDA: Data dependent acquisition
DNA: Deoxyribonucleic acid
FDR: False discovery rate
FPR: False positive rate
H: human
HPLC: High-performance liquid chromatography
LC-MS/MS: Liquid chromatography-tandem mass spectrometry
M: mouse
MOTU: molecular operational taxonomic unit
MS: Mass spectrometry
MS2: Tandem mass spectrometry
MS/MS: Tandem mass spectrometry
m/z: Mass-to-charge ratio
PSM: Peptid-to-spectrum match
R2: MOTU 2
R4: MOTU 4
TPR: True positive rate
Y: yeast
