# Supplementary material for: DISMS2: A flexible algorithm for direct proteome- wide distance calculation of LC-MS/MS runs
Source: BMC Bioinformatics. 2017 Mar 3;18:148. doi: 10.1186/s12859-017-1514-2 (PMC5335755; doi:10.1186/s12859-017-1514-2)
Supplement: Additional file 1 — Materials and Methods. This file provides detailed information about the sample preparation and LC-MS/MS analyses. (PDF 145 kb) [file 12859_2017_1514_MOESM1_ESM.pdf]

Materials and Methods

Materials

Cell lines

HeLa cell line (immortalized human cervix carcinoma) was obtained from DSMZ, Braunschweig, Germany (Article no: ACC161). Mouse cell line C2C12 (mouse CH3 muscle myoblast) was purchased from Sigma Aldrich, Steinheim, Germany (Article no: 91031101).

Chemicals and reagents

The following materials were purchased from Sigma-Aldrich, Steinheim, Germany: ammonium hydrogen carbonate (NH_4HCO_3), anhydrous magnesium chloride ($MgCl_2$), guanidine hydrochloride (GuHCl), iodoacetamide (IAA), and urea. Tris base was obtained from Applichem Biochemica, Darmstadt, Germany. Sodium dodecyl sulfate (SDS) was purchased from Carl Roth, Karlsruhe, Germany. Dithiothreitol (DTT), EDTA-free protease inhibitor (Complete Mini) tablets were bought from Roche Diagnostics, Mannheim, Germany. Sodium chloride (NaCl), calcium chloride ($CaCl_2$) and Benzonase were purchased from Merck, Darmstadt. Sequencing grade modified trypsin was bought from Promega, Madison, WI, USA. Bicinchoninic acid assay (BCA) kit was acquired from Thermo Fisher Scientific, Dreieich, Germany. All chemicals for ultra-pure HPLC solvents such as formic acid (FA), trifluoroacetic acid (TFA) and acetonitrile (ACN) were obtained from Biosolve, Valkenswaard, the Netherlands.

Methods

Cell, tissue and organism lysis

Unless otherwise stated the following procedures i.e. cell lysis, tissue dissection and ultrasonication were carried out on ice. Lysis and dissection procedures were performed under the laminar flow hood.

1. HeLa (*Homo sapiens*, H), C2C12 (*Mus musculus*, M), and Yeast-W303 (*Saccharomyces cerevisiae*, Y)

Approximately 1 mg of cells of each organism were subjected to 300 µL of lysis buffer (LB) comprising 50 mM Tris-HCl (pH 7.8), 150 mM NaCl, 1% SDS, and Complete Mini. Subsequently, 6 µL of benzonase (25 U/µL) and 2 mM $MgCl_2$ were added to the lysates and incubated at 37°C for 30 min. Next, the samples were clarified by centrifugation at 4°C and 18,000 g for 30 min.

2. Roundworm (*Caenorhabditis elegans*, strain N2 Bristol, wild type, C)

100 µL of washed *C. elegans* suspension was mixed with 500 µL of LB and the lysate was homogenized by ultrasonication for 30 seconds (amplitude: 30; pulse 1s/1s). The following steps i.e. benzonase treatment and centrifugation were performed as mentioned in 1.

3. Fruit fly (*Drosophila melanogaster*, wild type, D)

15 fruit flies were pooled and subjected to mechanical grinding using a mortar and pestle. To the ground material, 300 µL of LB were added and the following steps i.e. benzonase treatment and centrifugation were performed as mentioned in 1.

4. Fresh water snail, *Radix* species: MOTU2 (R2), MOTU4 (R4)

All *Radix* species belong to inbred lines and each snail was freshly harvested from an in house maintained aquarium and was immersed in ice cold ethanol (100%) for 10 min. Next, the foot was dissected using a sterile scalpel, thoroughly cleaned and cut into small pieces. To these, 300 µL of LB were added and subjected to mechanical grinding. Next, the lysates were further homogenized by ultrasonication for 30 seconds (amplitude: 30; pulse 1s/1s). Benzonase treatment and centrifugation were performed as mentioned in 1.

5. Foraminifera species: *Amphistegina lessonii* (Al), *Amphistegina gibbosa* (Ag)

As the entire foraminifera holobionts were analyzed, the samples contain the foraminiferal host as well as the endosymbiotic microalgae. Therefore, the foraminifera-symbiont association was treated together as a holobiont unit. Foraminifera were collected in the field (*A. gibbosa* in the Florida Keys and *A. lessonii* off the island of Zanzibar, Tanzania) and transported to Germany. Prior to analysis, they were incubated in sterile seawater for several days and brushed thoroughly to remove contaminations. 10 specimens of the same species were pooled and 100 µL of LB were added to them. Lysis was carried out by mechanical grinding. Next, the lysates were stored on ice for 30 min. Samples were clarified by centrifugation at 4°C and 10,000 g for 10 min.

Determination of protein concentration and carbamidomethylation

Estimation of protein concentration of the lysates was performed by a calorimetric bicinchoninic acid assay according to the manufacturers instructions (Pierce BCA Protein Assay Kit). In case of foraminifera lysates, the protein concentra-

tion was estimated based on reference samples of which the protein concentration was determined by amino acid analysis as previously described (1; 2). Next, cysteines were reduced by addition of 10 mM DTT at 56°C for 30 min followed by alkylation of free thiol groups with 30 mM IAA at room temperature (RT) in the dark for 30 min.

Sample clean up and proteolysis

Sample preparation and proteolysis were performed using filter-aided sample preparation (FASP) (3; 4) with minor changes. Briefly, cell lysate corresponding to 50 µg of protein (10 µg in case of foraminifera) was diluted 10-fold with freshly prepared 8.0 M urea/100 mM Tris-HCl (pH 8.5) buffer [5] and placed on the centrifugal device (PALL Nanosep, 30 kDa cutoff). The device was centrifuged at 13,500 g at RT for 30 min. All the following centrifugation steps were performed under the same conditions. To eliminate residual SDS, three washing steps were carried out with 100 µL of 8.0 M urea/100 mM Tris-HCl (pH 8.5). For buffer exchange, the device was washed thrice with 100 µL of 50 mM NH_4HCO_3 (pH 7.8). To the concentrated proteins, 100 µL of proteolysis buffer comprising of trypsin (Promega) (1:20 w/w ratio of protease to substrate), 0.2 M GuHCl and 2 mM $CaCl_2$ in 50 mM NH_4HCO_3 (pH 7.8), were added and incubated at 37°C for 14 h. The generated tryptic peptides were recovered by centrifugation with 50 µL of 50 mM NH_4HCO_3 followed by 50 µL of ultra-pure water. Finally, peptides were acidified by addition of 10% TFA (v/v) and digests were quality controlled as described previously [6].

LC-MS/MS analysis

At first each sample ($\sim$ 2 µL) was analyzed on a nano-LC-MS system. The sample amounts were corrected based on the alignment of total ion chromatograms (TICs) to compensate for systematic errors derived e.g. from the protein concentration estimation, such that each sample had identical amount of starting material prior to actual LC-MS analysis. All twenty seven samples (each 1 µg) were analyzed using an Ultimate 3000 nano RSLC system coupled to a Q Exactive mass spectrometer (both Thermo Scientific). Peptides were preconcentrated on a 100 µm x 2 cm C18 trapping column for 10 min using 0.1% TFA (v/v) with a flow rate of 20 µL/min followed by separation on a 75 µm x 50 cm C18 main column (both PepMap RSLC, Thermo Scientific) with a 120 min LC gradient ranging from 3-42% of buffer B: 84% acetonitrile, 0.1% formic acid at a flow rate of 250 nL/min. The Q Exactive HF MS was operated in data-dependent acquisition (DDA) mode and MS survey scans were acquired from m/z 300 to 1,500 at a resolution of 60,000 using the polysiloxane ion at m/z 371.101236 as lock mass [7]. Isolation of precursors was performed by the quadrupole with a window of 0.4 m/z. The fifteen (Top15) most intense signals were subjected to higher energy collisional dissociation (HCD) with a normalized collision energy (NCE) of 27% at a resolution of 15,000, taking into account a dynamic exclusion of 12 s. Automated gain control (AGC) target values were set to 3×10^6 for

MS and 5×10^4 MS/MS. Maximum injection times (IT) were 120 ms and 250 ms, respectively. Precursor ions with charge states of +1, > +5 or unassigned were excluded from MS/MS analysis. The "underfill" ratio, which specifies the minimum percentage of the target value likely to be reached at maximum fill time, was defined as 5%, which corresponds to a minimum precursor intensity of 2.5×10^3 to trigger a MS/MS scan.

Table M.1: Protein databases used for the respective samples; Decoy entries were generated automatically by Mascot.

Organism	Download details	Number of target sequences	Source
H. sapiens (H)	September 2014	20,162	`http://www.uniprot.org/`
S. cerevisiae (Y)	July 2015	6,622	`www.yeastgenome.org(SGD)`
M. musculus (M)	July 2015	16,746	`http://www.uniprot.org/`
C. elegans (C)	September 2015	26,596	`http://www.uniprot.org/`
D. melanogaster (D)	September 2015	22,005	`http://www.uniprot.org/`

References

[1] Cohen SA, Michaud DP. Synthesis of a Fluorescent Derivatizing Reagent, 6-Aminoquinolyl-N-Hydroxysuccinimidyl Carbamate, and Its Application for the Analysis of Hydrolysate Amino Acids via High-Performance Liquid Chromatography. Analytical Biochemistry. 1993; 211(2): 279–87.

[2] Shindo N, Nojima S, Fujimura T, Taka H, Mineki R, Murayama K. Separation of 18 6-Aminoquinolyl-carbamyl-Amino Acids by Ion-Pair Chromatography. Analytical Biochemistry. 1997; 249(1):79–82.

[3] Manza LL, Stamer SL, Ham AJ, Codreanu SG, Liebler DC. Sample preparation and digestion for proteomic analyses using spin filters. PROTEOMICS. 2005; 5(7):1742–5.

[4] Wisniewski JR, Zougman A, Nagaraj N, Mann M. Universal sample preparation method for proteome analysis. Nat Meth. 2009; 6(5):359–62.

[5] Kollipara L, Zahedi RP. Protein carbamylation: In vivo modification or in vitro artefact? PROTEOMICS. 2013; 13(6):941–4.

[6] Burkhart JM, Schumbrutzki C, Wortelkamp S, Sickmann A, Zahedi RP. Systematic and quantitative comparison of digest efficiency and specificity reveals the impact of trypsin quality on MS-based proteomics. Journal of Proteomics. 2012; 75(4):1454–62.

[7] Olsen JV, de Godoy LM, Li G, Macek B, Mortensen P, Pesch R, Makarov A, Lange O, Horning S, Mann M. Parts per Million Mass Accuracy on an Orbitrap Mass Spectrometer via Lock Mass Injection into a C-trap. Molecular & Cellular Proteomics. 2005; 4(12):2010–21.
