# Supplementary material for: DISMS2: A flexible algorithm for direct proteome- wide distance calculation of LC-MS/MS runs
Source: BMC Bioinformatics. 2017 Mar 3;18:148. doi: 10.1186/s12859-017-1514-2 (PMC5335755; doi:10.1186/s12859-017-1514-2)
Supplement: Additional file 2 — Table S1. Total number of MS/MS spectra in Table 4. (PDF 8 kb) [file 12859_2017_1514_MOESM2_ESM.pdf]

Comparison	Samples	Total number
C vs. C	C1, C2, C3	119813
D vs. D	D1, D2, D3	109930
H vs. H	H1, H2, H3	112075
M vs. M	M1, M2, M3	109887
Y vs. Y	Y1, Y2, Y3	108595
C vs. D	C1, C2, C3, D1, D2, D3	229743
C vs. H	C1, C2, C3, H1, H2, H3	231888
C vs. M	C1, C2, C3, M1, M2, M3	229700
C vs. Y	C1, C2, C3, Y1, Y2, Y3	228408
D vs. H	D1, D2, D3, H1, H2, H3	222005
D vs. M	D1, D2, D3, M1, M2, M3	219817
D vs. Y	D1, D2, D3, Y1, Y2, Y3	218525
H vs. M	H1, H2, H3, M1, M2, M3	221962
H vs. Y	H1, H2, H3, Y1, Y2, Y3	220670
M vs. Y	M1, M2, M3, Y1, Y2, Y3	218482
