# Supplementary material for: DISMS2: A flexible algorithm for direct proteome- wide distance calculation of LC-MS/MS runs
Source: BMC Bioinformatics. 2017 Mar 3;18:148. doi: 10.1186/s12859-017-1514-2 (PMC5335755; doi:10.1186/s12859-017-1514-2)
Supplement: Additional file 3 — Table S2. Standard Errors belonging to mean distances in Table 4. (PDF 9 kb) [file 12859_2017_1514_MOESM3_ESM.pdf]

Method	DB.ra	DB.ra.nodup	DISMS2.f	DB.a	DISMS2.af	DB.af
C vs. C	0.00114	0.00176	0.00164	0.00157	0.00133	0.00164
D vs. D	0.00537	0.00725	0.00663	0.00473	0.00448	0.00663
H vs. H	0.00220	0.00267	0.00155	0.00121	0.00109	0.00155
M vs. M	0.00241	0.00319	0.00363	0.00245	0.00338	0.00363
Y vs. Y	0.00188	0.00297	0.00254	0.00196	0.00253	0.00254
C vs. D	0.00038	0.00010	0.00167	0.00019	0.00070	0.00167
C vs. H	0.00026	0.00006	0.00065	0.00020	0.00031	0.00065
C vs. M	0.00016	0.00019	0.00178	0.00006	0.00077	0.00178
C vs. Y	0.00005	0.00007	0.00121	0.00004	0.00023	0.00121
D vs. H	0.00033	0.00023	0.00085	0.00017	0.00078	0.00085
D vs. M	0.00024	0.00016	0.00076	0.00009	0.00013	0.00076
D vs. Y	0.00017	0.00006	0.00036	0.00008	0.00022	0.00036
H vs. M	0.00079	0.00087	0.00138	0.00082	0.00161	0.00138
H vs. Y	0.00010	0.00009	0.00125	0.00007	0.00019	0.00125
M vs. Y	0.00012	0.00009	0.00050	0.00008	0.00022	0.00050
