# Supplementary material for: DISMS2: A flexible algorithm for direct proteome- wide distance calculation of LC-MS/MS runs
Source: BMC Bioinformatics. 2017 Mar 3;18:148. doi: 10.1186/s12859-017-1514-2 (PMC5335755; doi:10.1186/s12859-017-1514-2)
Supplement: Additional file 4 — Table S3. Additional information to Algorithm DB.af. Mean relative number of partners (same peptide), different peptides, missing annotation in list 1 and no remaining candidates after filtering in list 2 in Algorithm DB.af. (PDF 9 kb) [file 12859_2017_1514_MOESM4_ESM.pdf]

	Same peptide	Different peptide	List 1: Missing annotation	List 2: No remaining candidates
C vs. C	0.548	0.034	0.290	0.128
D vs. D	0.467	0.019	0.406	0.108
H vs. H	0.502	0.031	0.341	0.126
M vs. M	0.492	0.026	0.360	0.122
Y vs. Y	0.371	0.013	0.535	0.082
C vs. D	0.024	0.135	0.348	0.493
C vs. H	0.027	0.160	0.316	0.497
C vs. M	0.027	0.146	0.325	0.501
C vs. Y	0.007	0.114	0.412	0.466
D vs. H	0.031	0.132	0.373	0.464
D vs. M	0.033	0.122	0.383	0.462
D vs. Y	0.008	0.097	0.470	0.424
H vs. M	0.256	0.087	0.350	0.307
H vs. Y	0.009	0.112	0.438	0.441
M vs. Y	0.009	0.105	0.447	0.438
