# Supplementary material for: DISMS2: A flexible algorithm for direct proteome- wide distance calculation of LC-MS/MS runs
Source: BMC Bioinformatics. 2017 Mar 3;18:148. doi: 10.1186/s12859-017-1514-2 (PMC5335755; doi:10.1186/s12859-017-1514-2)
Supplement: Additional file 6 — Table S5. Coefficients of variation (CVs) of absolute differences of distances between two proteome comparisons methods. CVs of absolute differences of distances between two methods, within species (top) and between species (bottom), for the species roundworm (C), fruit fly (D), human (H), mouse (M), and yeast (Y). Values smaller than 0.5 indicate relevant differences between the corresponding method pairs. Values greater than 0.5 are marked (*). (PDF 9 kb) [file 12859_2017_1514_MOESM6_ESM.pdf]

Method A vs. Method B	DISMS2.af<br>DB.af	DISMS2.af<br>DISMS2.f	DISMS2.f<br>DB.ra	DISMS2.f<br>DB.ra.nodup	DB.a<br>DISMS2.f
C vs. C	0.4448	0.0047	0.0065	0.0062	0.0031
D vs. D	0.2715	0.0156	0.0193	0.0270	0.0235
H vs. H	0.9901*	0.0224	0.0161	0.0309	0.0277
M vs. M	0.3774	0.0056	0.0140	0.0111	0.0261
Y vs. Y	0.6739*	0.0080	0.0072	0.0143	0.0037
C vs. D	0.1631	0.1949	0.4332	0.1568	0.1841
C vs. H	0.0404	0.0794	0.0884	0.0491	0.0555
C vs. M	0.1614	0.2436	0.5458*	0.1774	0.2142
C vs. Y	0.0952	0.4221	0.2509	0.1832	0.1779
D vs. H	0.1246	0.0630	0.1267	0.0512	0.0610
D vs. M	0.0212	0.0753	0.1360	0.0499	0.0595
D vs. Y	0.0597	0.0657	0.0337	0.0330	0.0303
H vs. M	0.8931*	0.0303	0.0389	16.6491*	0.0242
H vs. Y	0.0519	0.1780	0.1468	0.1111	0.1134
M vs. Y	0.0557	0.0918	0.0781	0.0560	0.0552
