# Supplementary figures and images for: DISMS2: A flexible algorithm for direct proteome- wide distance calculation of LC-MS/MS runs
Source: BMC Bioinformatics. 2017 Mar 3;18:148. doi: 10.1186/s12859-017-1514-2 (PMC5335755; doi:10.1186/s12859-017-1514-2)

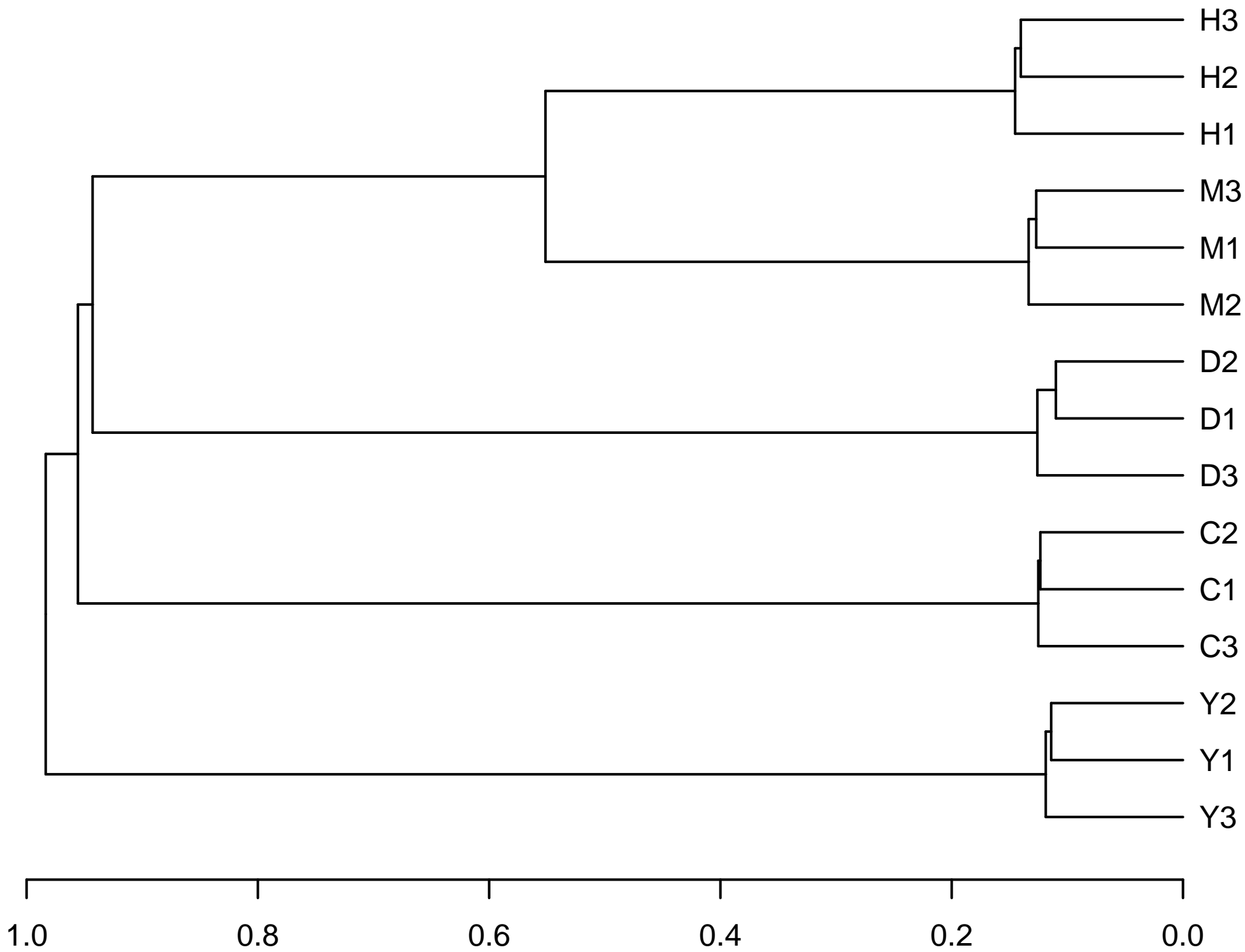

Supplement: Additional file 7 — Figure S1. Dendrogram for three technical replicates each of roundworm (C), fruit fly (D), human (H), mouse (M) and yeast (Y) using average linkage hierarchical clustering based on all pairwise distances of 15 MS/MS runs. Computed via method DB.ra. (PDF 4 kb) [file 12859_2017_1514_MOESM7_ESM.pdf]

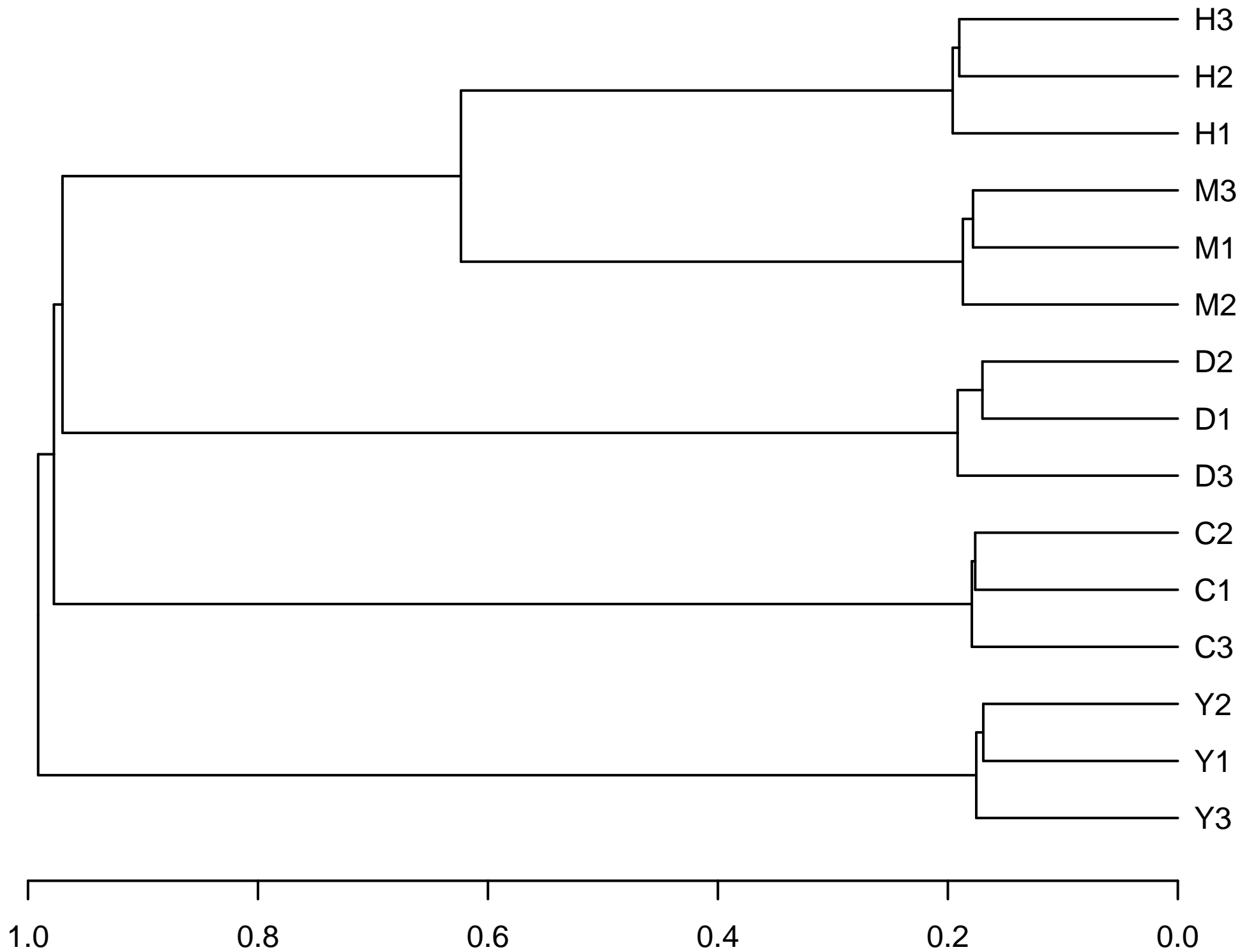

Supplement: Additional file 8 — Figure S2. Dendrogram for three technical replicates each of roundworm (C), fruit fly (D), human (H), mouse (M) and yeast (Y) using average linkage hierarchical clustering based on all pairwise distances of 15 MS/MS runs. Computed via method DB.ra.nodup. (PDF 4 kb) [file 12859_2017_1514_MOESM8_ESM.pdf]

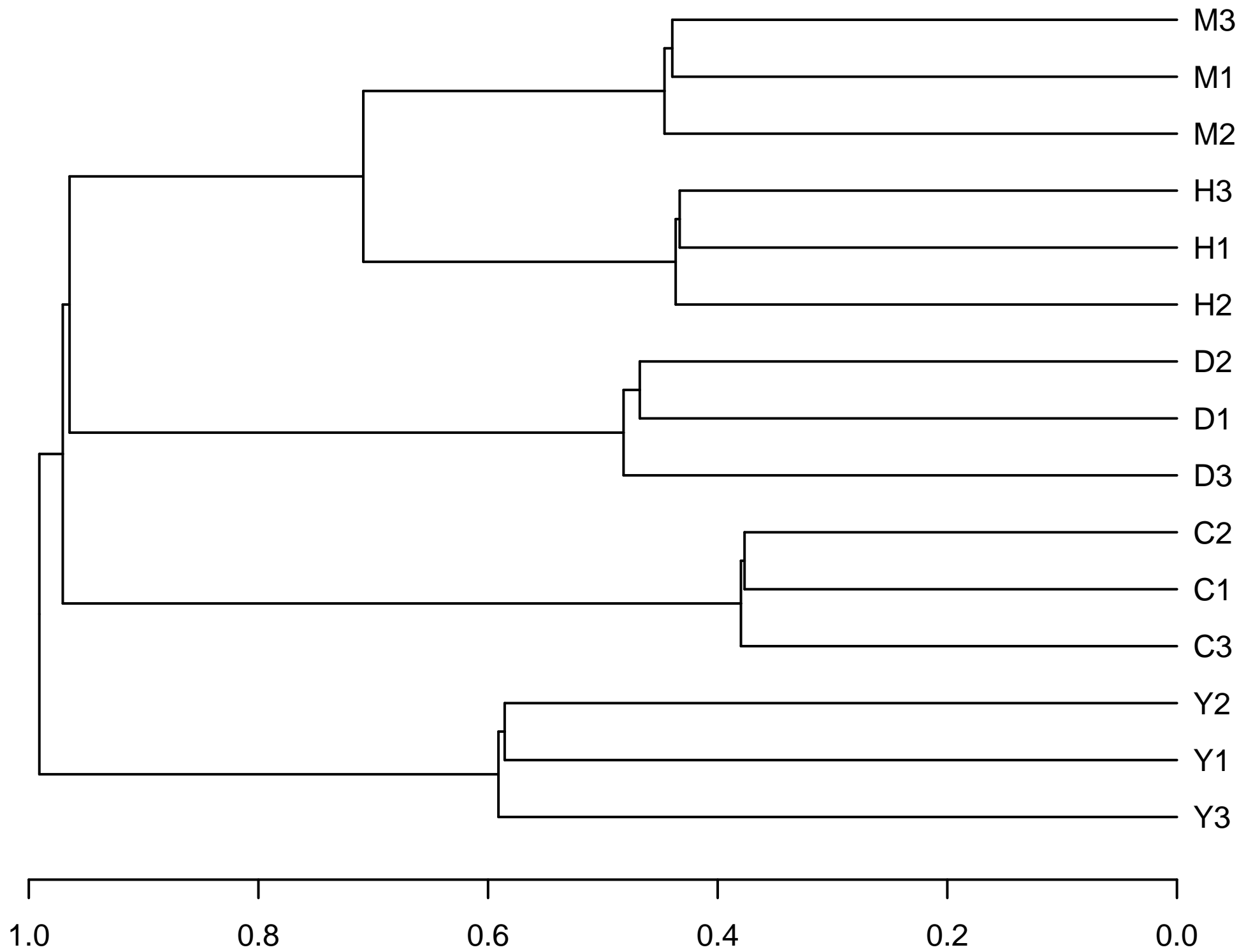

Supplement: Additional file 9 — Figure S3. Dendrogram for three technical replicates each of roundworm (C), fruit fly (D), human (H), mouse (M) and yeast (Y) using average linkage hierarchical clustering based on all pairwise distances of 15 MS/MS runs. Computed via method DB.a. (PDF 4 kb) [file 12859_2017_1514_MOESM9_ESM.pdf]

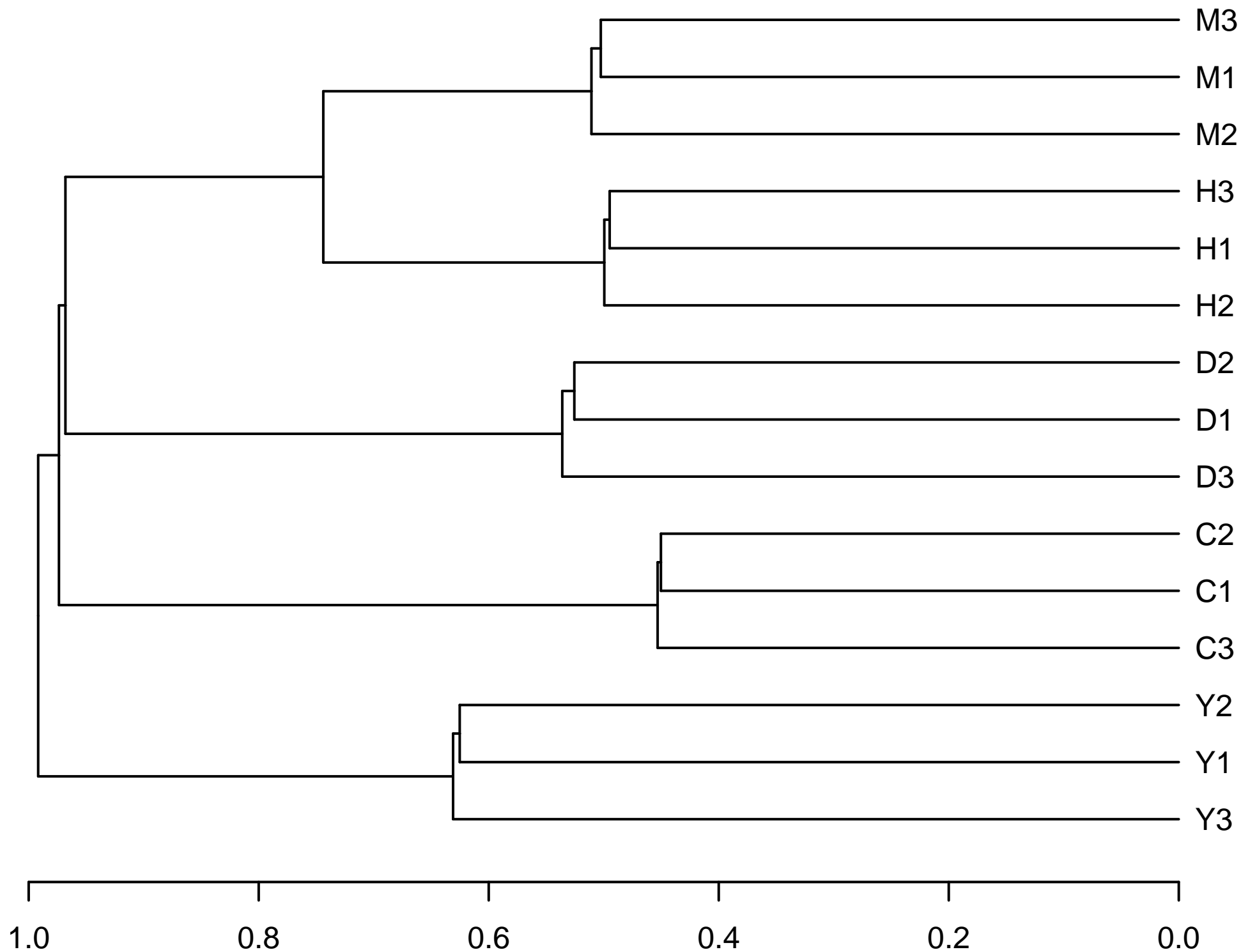

Supplement: Additional file 10 — Figure S4. Dendrogram for three technical replicates each of roundworm (C), fruit fly (D), human (H), mouse (M) and yeast (Y) using average linkage hierarchical clustering based on all pairwise distances of 15 MS/MS runs. Computed via method DB.af. (PDF 4 kb) [file 12859_2017_1514_MOESM10_ESM.pdf]
